# Accuracy of the smartphone blood pressure measurement solution OptiBP to track blood pressure changes in pregnant women

**DOI:** 10.1097/HJH.0000000000003956

**Published:** 2025-02-07

**Authors:** Pedro Almeida, Alexia Cuénoud, Harry Hoang, Alexandra Othenin-Girard, Nadia Salhi, Andreas Köthe, Urvan Christen, Patrick Schoettker

**Affiliations:** a Department of Anesthesiology, Lausanne University Hospital; b University of Lausanne; c Biospectal SA, Lausanne, Switzerland

**Keywords:** application, blood pressure, cuffless, optical signal, pregnancy, smartphone

## Abstract

**Introduction:**

Hypertensive disorders present significant morbidity and mortality during pregnancy. Although ambulatory blood pressure measurement remains the standard of care for normotensive women, self-monitoring at home is increasingly prevalent. The widespread use of smartphones worldwide has sparked interest in mobile applications that leverage the built-in hardware for blood pressure estimation, yet few trials have assessed their accuracy.

**Methods:**

This prospective, longitudinal and monocentric study evaluated the accuracy of the OptiBP algorithm against standard oscillometric blood pressure measurements in a sample of pregnant women. Patients scheduled for elective caesarean sections were enrolled during the preoperative anesthesia consultations. Paired blood pressure measurements using OptiBP and the reference method were obtained at multiple time-points in late pregnancy and the postpartum period. Agreement between methods was assessed using the AAMI/ESH/ISO 81060-2:2018 standard thresholds of 5 ± 8 mmHg for mean ± standard deviation of the error (criterion 1) and patient-specific standard deviation of the mean error (criterion 2) and represented graphically by Bland–Altman scatterplots.

**Results:**

Forty-eight women were enrolled of which 32 completed the protocol, yielding 338 total valid measurement pairs. Mean and standard deviation of the error were −1.78 ± 7.94 and 1.19 ± 7.59, and the patient-specific standard deviation of the mean error was 4.68 and 4.52, for SBP and DBP, respectively.

**Conclusion:**

Compared with blood pressure measurements taken with an oscillometric device, OptiBP's blood pressure estimates meet the AAMI/ESH/ISO 81060-2:2018 criteria.

## Graphical abstract

**Figure:**
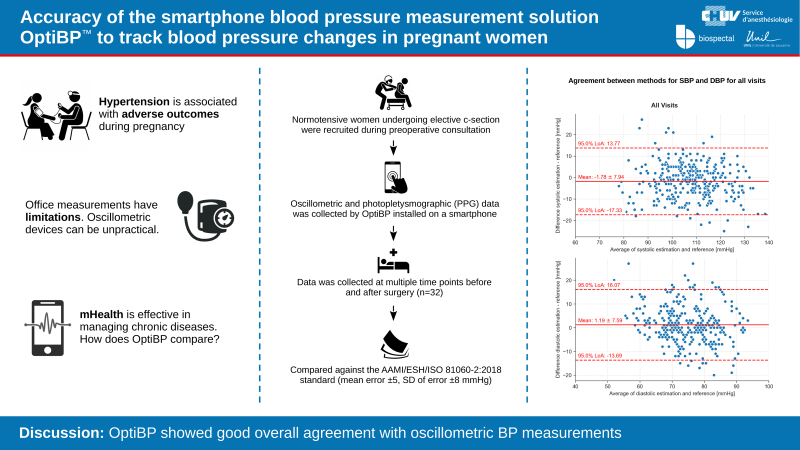

## INTRODUCTION

Adverse changes in blood pressure (BP) during gestation are a well documented phenomenon [1]. Hypertension affects 14% of pregnancies [2] and accounts for 10% of all causes of maternal death [3], more than half of which are preventable [4]. It also carries significant morbidity for both mother and newborn [5].

Although most guidelines recommend monitoring BP at each prenatal consultation [6], periodic measurements provide limited information due to the widespread prevalence of white coat [7] and masked hypertension [8], and the oftentimes unpredictable and life-threatening nature of hypertensive disorders of pregnancy (HDP) [9].

Home BP monitoring (HBPM) is recommended for the general hypertensive population and has been shown to be more accurate than office monitoring and a better predictor of cardiovascular disease burden [10]. During pregnancy, HBPM is recommended for individuals with gestational and chronic hypertension, especially when uncontrolled [11,12], and emerging evidence suggests it is a reliable alternative that might involve fewer antenatal visits [13,14].

Although oscillometric devices remain a popular choice – used by as many as 49% of pregnant women for self-monitoring [15] – they can be costly and not every consumer device has undergone validation trials [16,17].

Concurrently, mobile health technologies fueled by the widespread use of smartphones [18] have proven effective in managing chronic diseases [19]. Advances in technology and miniaturization have led to the development of apps that leverage the phone's hardware, often using photoplethysmographic (PPG) signals acquired by the camera's sensor that are used to directly estimate BP [20].

A review of 107 mobile apps dedicated to hypertension found that 14% claimed to measure BP natively, despite a lack of transparency concerning methodology or medical and regulatory oversight [21]. Moreover, formal assessments in clinical settings have shown accuracy to be low [22], including in pregnant women [23].

Recently, OptiBP, a dedicated blood pressure mobile app developed by a Swiss startup [24], was found to be accurate in accordance with the AAMI/ESH/ISO 81060-2:2018 standard [25,26] in a group of hypertensive patients in an ambulatory setting [27,28], in more than 700 patients in low-income countries [29] and in patients in the postanesthetic care unit compared with a noninvasive automatic oscillometric device [30]. Nevertheless, accuracy needs to be demonstrated in various demographics. As such, the present study aims to evaluate OptiBP's performance in a cohort of pregnant women.

## METHODS

### Study design

This was a prospective, longitudinal and monocentric study assessing OptiBP performance in estimating BP changes from reference values obtained with a noninvasive oscillometric device in gravid women at various timepoints of their pregnancy and postpartum period.

The study protocol was published in www.clinicaltrials.gov and www.kofam.ch (NCT03875248) on 14 March 2019 and includes various amendments.

SwissEthics CER-VD (BASEC 2018-01656) and Swissmedic provided ethical and regulatory approval, respectively. The trial was conducted in compliance with European Directive 93/42/EEC concerning medical devices, the Swiss Ordinance on clinical trials of medical devices (ClinO-MD, C2) and the ISO 14155:2020 international standard. All patients gave written informed consent.

### Study population

Pregnant women scheduled for a preoperative anesthesia consultation in preparation for an elective cesarean section were screened and recruited at the maternity hospital at Lausanne University Hospital (Lausanne, Vaud, Switzerland) between September 2022 and December 2023.

Inclusion criteria were individuals 18 years or older, conversational fluency in the trial language (French) and capacity to provide written informed consent.

Exclusion criteria were women with currently diagnosed gestational hypertension or preeclampsia; known therapeutic compliance issues including substance abuse and neuropsychiatric disorders; a BP difference greater than 15 mmHg systolic and/or more than 10 mmHg diastolic between arms, as determined by the average of the difference of three sets of alternating arm BP measurements using the reference device; recent severe cardiopulmonary disease, such as acute myocardial infarction, pulmonary embolism or decompensated heart failure; dysrhythmias; hand disability or deformity that would hinder proper use of the smartphone camera for signal acquisition; and known contact dermatitis to nickel and/or chromium.

### Protocol

Following enrolment, patients underwent app calibration (visit 0), by inputting age, weight and height into CamBP v3.0.4(4), the double-blinded, clinical trial version equivalent of OptiBP app. Baseline BP values were taken twice with an oscillometric reference device (M3 Comfort, OMRON Healthcare, Kyoto, Japan) and then twice with the app [installed on two Samsung S21 5G (SM-G991B/DS), Seoul, South Korea].

**FIGURE 1:**
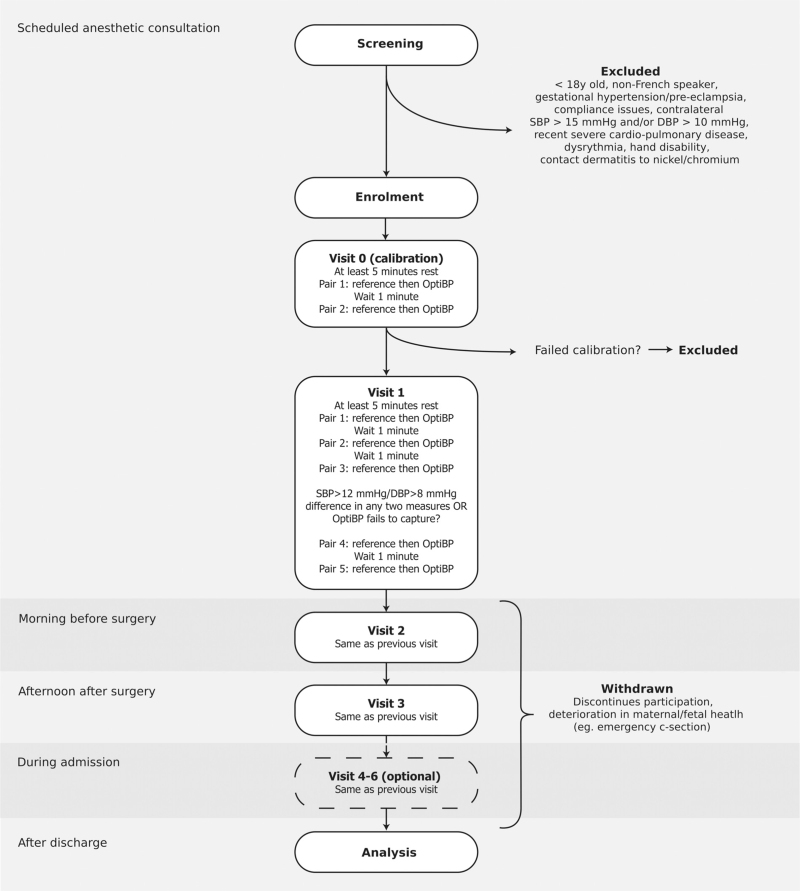
Trial protocol flowchart.

Subsequently, patients underwent three visits (visits 1, 2 and 3) during which three pairs of BP measurements (pair 1, 2 and 3) were taken (Fig. 1). A pair was defined as a measurement using the reference device on the left arm immediately followed by a PPG signal acquisition with CamBP on the right arm. For each pair, a maximum of two attempts at capturing PPG signals were performed by CamBP. If both failed, the researcher collecting data would move to the next measurement pair. Each visit started with at least a 5 min rest period either sitting or in the supine position with a 45° bed inclination, followed by at least 1 min of pause between each pair of measurements.

Up to two additional measurement pairs (pair 4 and 5) were collected in the following situations: any two of the previous pairs showed a difference exceeding 12 mmHg systolic and/or 8 mmHg diastolic with the reference device; or if CamBP failed to capture data twice consecutively in any one pair.

Each protocol visit took place at distinct stages of the pregnancy: immediately after calibration (visit 1), on the morning of the scheduled cesarean section, in the labor ward prior to being transferred to the operating room (visit 2) and after the cesarean section when the patient had returned to her room in the maternity ward (visit 3). In addition, and at the discretion of the research team and availability of the patient, up to three daily additional visits (visits 4, 5, and 6) were scheduled during hospital stay to expand the data.

Patients were prematurely withdrawn from the trial under the following conditions: failure to meet the quality criteria for signal acquisition during calibration (failed calibration), any deterioration in maternal and/or fetal health that prevented follow-up visits (e.g. an emergency cesarean section) or an individual decision to discontinue participation.

### Data acquisition, processing and analysis

Data collection, OptiBP algorithm and its integration with the mobile app has been discussed in greater detail in previous publications [27,31–33]. In summary, light emitted by the smartphone LED flash traverses the fingertip resting against the camera array, refracts along tissue and is captured by the optical sensor. OptiBP then records short, high-speed video sequences of PPG signals that translate into volumetric changes in blood flow. Each PPG signal sequence is assigned a quality index and then passes through a bank of time-derived filters that characterize morphological variations at different temporal resolutions. Lastly, the algorithm estimates nominal changes in BP relative to an arbitrary baseline, determined during calibration. All data processing and BP estimations were done offsite and blinded to the study team.

### Outcomes

The primary outcome was the agreement between estimated SBP and DBP values using OptiBP and paired measurements with the reference device.

Secondary outcomes were OptiBP's performance in capturing PPG signals, defined as the rate of successful measurement attempts, as well as adverse events.

### Sample size

The AAMI/ESH/ISO 81060-2:2018 standard recommends including at least 30 participants when studying a pregnant population with a previously validated sphygmomanometer, subdivided into two equal groups of normotensive and preeclamptic patients each [26]. We recruited normotensive patients until 30 had completed protocol.

### Statistical analysis

Primary analysis evaluated the agreement between OptiBP and the reference method, framed against AAMI/ESH/ISO 81060-2:2018 standard pass criteria 1 and 2 [25,26].

For SBP and DBP, the mean and standard deviation (SD) of the error, defined as the difference between OptiBP and reference device measurements, were calculated. These metrics were then compared with the standard's validation thresholds: a mean error of ±5 mmHg or less and SD of the error of 8 mmHg or less (criterion 1).

Then, the patient-specific SD of the mean error (criterion 2) was determined. This involved averaging each patient's BP measurements and calculating the SD of the difference between those averages as obtained from OptiBP and the reference device. These values were compared against the maximum permissible SD as a function of the initially estimated mean error, as per the standard's predefined values.

Finally, Bland–Altmann plots [34] were utilized to visually depict mean error (bias) ± SD, along with 95% limits of agreement (mean error ± 1.96 × SD).

The analysis and its graphical representations were executed using Python v3.10 (Python Software Foundation, Delaware, USA) running pandas, numpy and matplotlib libraries. The source-code was edited using Visual Studio Code v1.74.1 (Microsoft Corporation, Redmond, Washington, USA). Additional media were designed in Inkscape 1.3.2 (Free Software Foundation Inc, Boston, Massachusetts, USA).

## RESULTS

**FIGURE 2:**
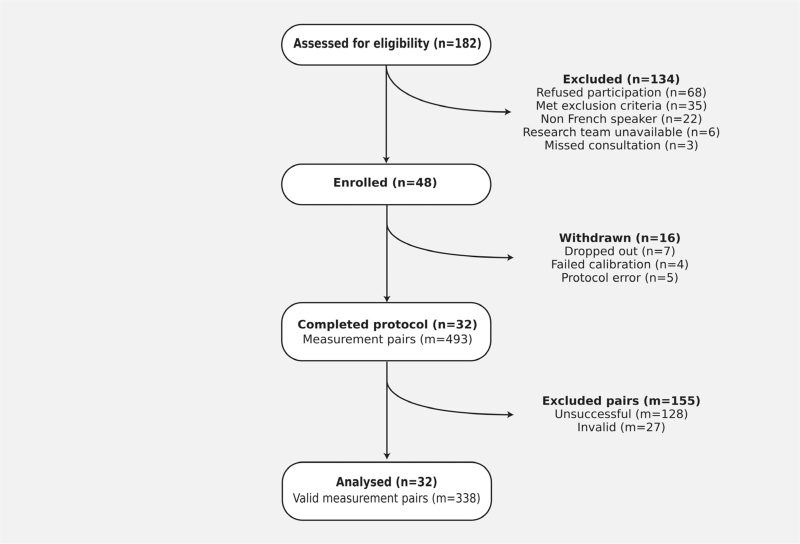
Modified CONSORT flowchart. Unsuccessful measurements are attempts where OptiBP failed to capture a PPG signal. Invalid measurements are attempts where the captured PPG signal was not usable to generate a BP estimation.

Of the 48 patients enrolled, 32 completed the protocol (Fig. 2). Sixteen patients were excluded: seven dropped out (four underwent emergency c-section and three had their elective c-section performed ahead of schedule), four failed calibrations, and five in whom the protocol was applied incorrectly. This translated into 365 successful BP measurement pairs, 338 of which were deemed valid by OptiBP's algorithm. Baseline patient's characteristics are presented in Table 1.

**TABLE 1:** Baseline characteristics

Variables	(*N* = 32)
Age (years)	35.00 ± 4.19
Height (cm)	165.09 ± 5.97
Weight (kg)	79.21 ± 11,70
BMI (kg/m^2^)	28.78 ± 4.51
Weeks of gestation	38 [37–38]
Previous HDP	2 (6.25%)
Reason for c-section^a^	
Maternal request	10 (29.41%)
Abnormal fetal presentation	9 (26.47%)
Maternal medical condition	8 (23.53%)
Previous c-section	5 (14.71%)
Macrosomia	1 (2.94%)
Fetal pathology	1 (2.94%)

Variables are presented as mean ± SD or median [Q1–Q3], according to normality assessed using Shapiro–Wilk test, or absolute number (percentage). HDP, hypertensive disorders of pregnancy.

a *n* = 34 as two patients checked two reasons for c-section in a prespecified inventory.

**FIGURE 3:**
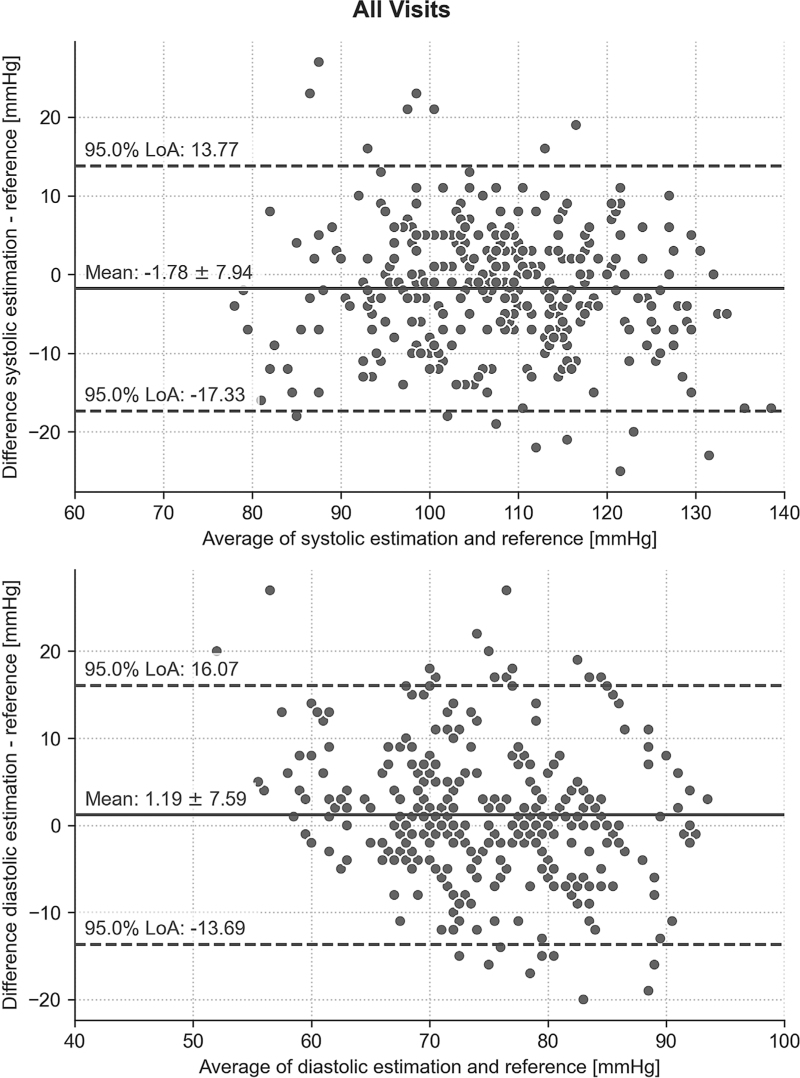
Bland–Altman plots with agreement between OptiBP and reference method for SBP and DBP, for all visits.

The agreement between methods, for all visits, for SBP exhibited a mean error ± SD of −1.78 ± 7.94 and an average SD of the mean error of 4.68 with maximum permissible SD of 6.71. For DBP, the mean error ± SD was 1.19 ± 7.59 and the average SD of the mean error was 4.52, with a maximum permissible SD of 6.84 (Table 2). OptiBP's overall success rate in capturing data was 79% across all visits. Additionally, Bland–Altmann plots are shown for all visits in Fig. 3. Bland–Altmann plots for individual visits are available as supplement (Graph, Supplemental Digital Content 1 and 2), alongside graphical distribution of blood pressure for all and individual visits (Graph, Supplemental Digital Content 3). No adverse events were registered.

**TABLE 2:** Results for aggregate visits and individual visits

Visit	*N*	SBP		DBP		Measurements		
		Criterion 1	Criterion 2	Criterion 1	Criterion 2	Attempted	Successful (%)	Valid (%)
All	32	−1.78 ± 7.94	4.68 (6.71)	1.19 ± 7.59	4.52 (6.84)	463	365 (79)	338 (93)
1	32	−0.55 ± 7.21	5.42 (6.91)	0.36 ± 4.52	3.20 (6.93)	110	101 (92)	43 (90)
2	32	−5.72 ± 7.09	6.27 (4.79)	−4.73 ± 5.92	5.92 (5.08)	120	88 (73)	33 (89)
3	32	−1.53 ± 8.8	8.34 (6.78)	5.15 ± 7.48	8.10 (4.79)	118	84 (71)	81 (96)
4	22	0.84 ± 8.02	7.26 (6.89)	4.72 ± 8.14	7.77 (5.08)	80	68 (85)	64 (94)
5	7	−2.56 ± 4.98	4.35 (6.43)	2.33 ± 8.03	7.87 (6.55)	27	18 (67)	18 (100)
6	2	1.67 ± 5.20	1.41 (6.43)	−3.67 ± 4.55	0.47 (5.83)	8	6 (75)	6 (100)

For SBP and DBP, criterion 1 expressed as mean ± SD and criterion 2 as average SD of the mean error (maximum permissible SD of the mean error). Measurements are represented by attempted, successful (percentage over attempted) and valid (percentage over successful).

## DISCUSSION

Consistent with earlier publications [27,28,30,35,36], our study shows strong agreement between OptiBP-derived BP values and those obtained via the reference method.

To the best of our knowledge, this is the first study evaluating a cuffless optical blood pressure solution in the form of a smartphone embedded app in a dedicated cohort of women at various timepoints during their pregnancy.

Raichle *et al.*[23] evaluated an iOS app in 32 pregnant women, including 1 with preeclampsia. They found it to overestimate BP when SBP was less than 130 mmHg and underestimate when it was 130–160 mmHg, yielding a mean error ± SD of 5 ± 14.5 mmHg when compared to a reference automatic oscillometric device.

A trial by Ryu *et al.*[37], employing a custom sensor array to measure biometric data from pregnant women, collected PPG signals from the chest and finger to calculate Pulse Arrival Time (PAT) as a surrogate for BP. Although they found good agreement with a traditional sphygmomanometer, with a mean error ± SD of 0.4 ± 7.8 mmHg, the trial was limited to three healthy participants.

Festo *et al.*[29] published an independent, multisite trial, assessing OptiBP's performance in three emergent economies and found it to meet AAMI/ESH/ISO 81060-2:2018 criteria both overall and in their subgroup analysis of over 150 pregnant women from a tertiary hospital in South Africa. Although they included a significant subset of patients with gestational hypertension and preeclampsia, theirs involved only one set of measurements taken shortly after calibration.

It is generally accepted that BP decreases in the early stages of pregnancy, with systemic vasodilation starting as early as 5 weeks and peaking in the second trimester before returning to near pregravid levels at the end of the pregnancy [38]. Conversely, cardiac output and peripheral vascular resistance remain respectively high and low into the second week of the postpartum period [39]. In addition, perioperative hemodynamic changes are frequent during caesarean section because of the predictable blood loss [40] and the decreased sympathetic drive attributed to spinal anesthesia [41].

Despite these observations, the longitudinal data collected in this study showed minimal BP variability. This may be explained by two factors: first, most women were enrolled in the tail end of their pregnancy where physiological changes in BP are less pronounced; second, the sympathetic blockade and intravascular volume depletion due to intraoperative bleeding were already resolved at the moment of assessment and did not translate into significant BP changes.

Finally, while the evidence favors the use of HBPM for women with known chronic hypertension or HDP, it is unclear if it might facilitate new diagnoses or affect maternal and perinatal outcomes in otherwise healthy patients. In a sample of pregnant women in the UK, the BUMP1 trial found that self-monitoring in patients at risk of preeclampsia did not shorten the time to clinic-based detection of hypertension compared with standard care, which entailed a minimum of seven antenatal visits [42]. Importantly, 27% of the randomized women were already self-monitoring, potentially diluting the impact of the intervention, and the applicability of those findings to populations with varying healthcare access is yet to be determined.

### Limitations

Our study presents several limitations. Although meeting the AAMI/ESH/ISO 81060-2:2018 standard for the aggregate of visits, it marginally fails in visits 2 through 6. Despite being the de facto benchmark for cuffless designs, the norm was originally created for intermittent noninvasive automated devices using a cuff, which operate by measuring absolute values of arterial pressure. In contrast, OptiBP and other PPG-based algorithms work by estimating values relative to an arbitrary, predetermined baseline (i.e. the calibration). As the estimates naturally gravitate around this baseline, the mean of the measurements may reflect the calibration of the reference method rather than the estimates of the device itself. Repeated measurements compound this issue by flattening physiological variability, potentially leading to a false sense of precision [43].

To address these issues, alternative standards have been proposed. The IEEE 1708-2019a standard [44], was the first recognizing the particularities of cuffless devices. Although the standard stipulates that measurements must occur in static conditions following predefined BP changes, it falls short of detailing reproducible methods to induce these changes.

The ISO 81060-3:2022 [45] introduced a new norm for cuffless, continuous devices but its scope is limited to designs with BP output periods of 30 s or less, which is not representative of most mass-market cuffless devices. Furthermore, this standard was envisioned for clinical environments like operating theaters or ICUs where BP changes have direct therapeutic implications and where reference values are obtained via arterial catheterization.

Recently, the ESH Working Group on Blood Pressure Monitoring and Cardiovascular Variability released a set of recommendations aimed at validating intermittent cuffless devices like OptiBP [46]. The guidelines consist of various static and dynamics tests, designed to be compatible with different types of devices based on their function and calibration method, and employing the same pass criteria as those outlined in the ISO 81060-2:2018 standard. Although this represents the most comprehensive framework for evaluating consumer-oriented cuffless devices, it is unclear if the model can be applied as is to special populations, including pregnant women.

Second limitation, the 38% refusal rate among eligible participants may have introduced selection bias, even though OptiBP's performance is unlikely to be influenced by this factor. The main reasons for refusal were reluctance to complicate existing childbirth plans or lack of interest in the study question. The exclusion of women with known chronic hypertension or HDP, who are the most likely beneficiaries of self-monitoring, amplifies this bias. Although required by the AAMI/ESH/ISO 81060-2:2018 for validation in this population, the exclusion was a conscious decision owing to the study's proof-of-concept nature and aim for a homogeneous participant sample. We also recognize that most failed measurement attempts occurred during the second visit in the labor ward and the third visit in the patient's room in the hours following surgery, which might point to an underlying physiological mechanism. Indeed, both clinical experience and existing literature suggest that expectant surgical patients often experience peripheral vasoconstriction associated with higher anxiety [47], which might have impaired OptiBP's ability to capture PPG signals. Consistently, earlier evaluations of OptiBP in the perioperative setting showed similar fail rates, particularly among patients receiving vasopressive medication [30,36]. We would also address the low count of visits 5 and 6, as these were performed on postoperative day 2 and 3 respectively, when most patients had already been discharged.

Third, we acknowledge the lack of consideration for racial diversity in our sample. Pulse oximetry has been shown to have lower accuracy in hypoxemic, dark-skinned patients [48], with potential implications for healthcare [49], and OptiBP technology might suffer from similar biases, given the shared underlying physical principles. However, Festo *et al.*'s trial [29] suggests that OptiBP delivers accurate readings across ethnically diverse populations.

Lastly, almost 10% of the patients experienced calibration failure which might point to the existence of a hitherto unidentified subset of patients for whom OptiBP is ineffective at capturing signal. Given the body of evidence supporting OptiBP's performance and the nature of a new technology, we judge it to be due to user error in app and phone handling.

In conclusion, our study shows that OptiBP meets AAMI/ESH/ISO 81060-2:2018 criteria and can reliably produce BP estimates in pregnant women that are comparable to standard oscillometric methods. Future research should aim to include a diverse sample and focus on participants with diagnosed hypertensive disorders of pregnancy.

## ACKNOWLEDGEMENTS

We would like to acknowledge the help and support of Chloé Stoll in launching the trial; Jean-François Knebel and Salil Apte in analyzing and preparing data; and Nicolas Gurtner, Cyril Ben-Amara and Mélanie Boand in collecting data.

Previous presentation: preliminary data presented at the 33rd European Meeting on Hypertension and Cardiovascular Protection and published in the *Journal of Hypertension* 42 (Suppl 1): e88, May 2024.

Disclosures: Biospectal SA provided two smartphones preinstalled with OptiBP app and one automatic sphygmomanometer for the duration of the study. Biospectal SA had no influence on data collection but was responsible for analyzing raw PPG data and providing statistical analysis. OptiBP has secured the European CE MDR Class IIa mark for medical device certification.

### Conflicts of interest

P.S. serves as Chief Medical Advisor and A.K. as Head of R&D at Biospectal SA. U.C. was lead R&D Scientist at Biospectal SA during the trial period.

## Supplementary Material

Supplemental Digital Content

## Supplementary Material

Supplemental Digital Content

## Supplementary Material

Supplemental Digital Content
