# Supplementary material for: Accuracy of the smartphone blood pressure measurement solution OptiBP to track blood pressure changes in pregnant women
Source: J Hypertens. 2025 Feb 7;43(4):665–72. doi: 10.1097/HJH.0000000000003956 (PMC11872272; doi:10.1097/HJH.0000000000003956)

**Figure 2:** Bland-Altman plots with agreement between OptiBP and reference method for DBP and individual visits.


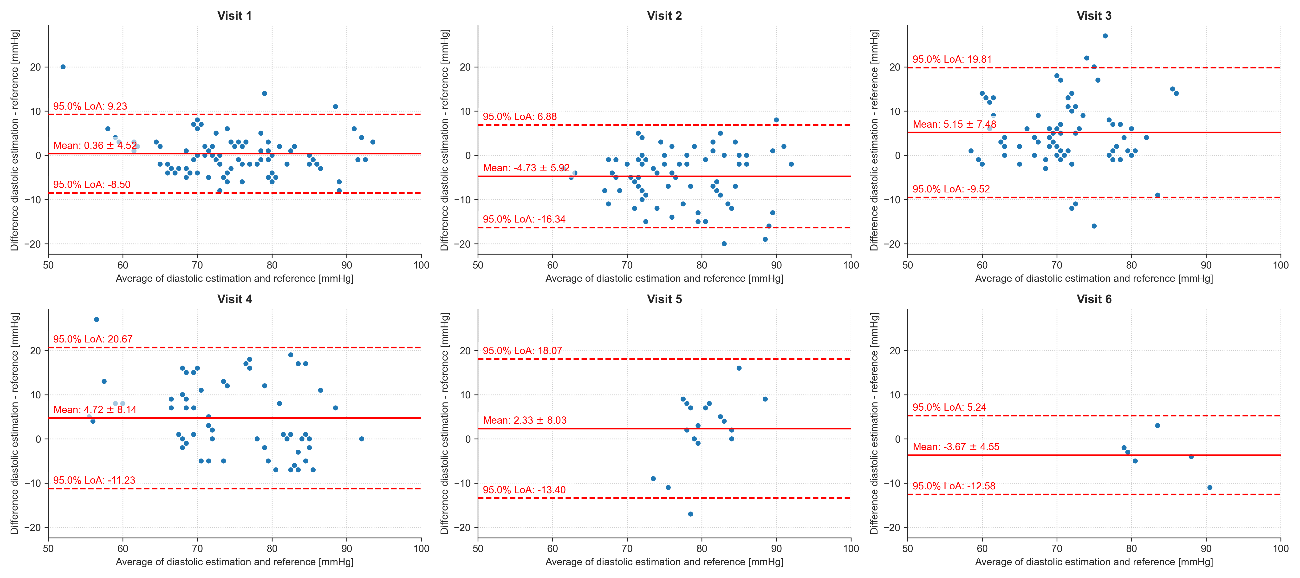

Supplement: Supplemental Digital Content [file jhype-43-665-s002.docx]
